# A Low-FODMAP Diet for Irritable Bowel Syndrome: Some Answers to the Doubts from a Long-Term Follow-Up

**DOI:** 10.3390/nu12082360

**Published:** 2020-08-07

**Authors:** Massimo Bellini, Sara Tonarelli, Federico Barracca, Riccardo Morganti, Andrea Pancetti, Lorenzo Bertani, Nicola de Bortoli, Francesco Costa, Marta Mosca, Santino Marchi, Alessandra Rossi

**Affiliations:** 1 Gastrointestinal Unit–Department of Translational Sciences and New Technologies in Medicine and Surgery, University of Pisa, 56124 Pisa, Italy; massimo.bellini@med.unipi.it (M.B.); barracca.federico@gmail.com (F.B.); pancio10@alice.it (A.P.); lorenzobertani@gmail.com (L.B.); nicola.debortoli@unipi.it (N.d.B.); fcosta@med.unipi.it (F.C.); santino.marchi@unipi.it (S.M.); 2 SOD Clinical Trial Statistical Support, Azienda Ospedaliero Universitaria Pisana, 56126 Pisa, Italy; r.morganti@ao-pisa.toscana.it; 3 Clinical and Experimental Medicine–Rheumatology Unit, University of Pisa, 56100 Pisa, Italy; marta.mosca@med.unipi.it (M.M.); alessandra.rossi@unipi.it (A.R.)

**Keywords:** irritable bowel syndrome, bowel habits, FODMAP, low-FODMAP diet, nutritionist, bioelectrical impedance

## Abstract

A low-FODMAP (fermentable oligosaccharides, disaccharides, monosaccharides and polyols) diet (LFD) is a possible therapy for irritable bowel syndrome (IBS). This study investigates the short- and long-term efficacy and nutritional adequacy of an LFD and the patients’ long-term acceptability. Patients’ adherence and ability to perceive the “trigger” foods were also evaluated. Seventy-three IBS patients were given an LFD (T0) and after 2 months (T1), 68 started the reintroduction phase. At the end of this period (T2), 59 were advised to go on an Adapted Low-FODMAP Diet (AdLFD) and 41 were evaluated again after a 6–24 month follow-up (T3). At each time, questionnaires and Biolectrical Impedance Vector Analysis (BIVA) were performed. The LFD was effective in controlling digestive symptoms both in the short- and long-term, and in improving quality of life, anxiety and depression, even if some problems regarding acceptability were reported and adherence decreased in the long term. The LFD improved the food-related quality of life without affecting nutritional adequacy. When data collected at T0 were compared with those collected at T2, the perception of trigger foods was quite different. Even if some problems of acceptability and adherence are reported, an LFD is nutritionally adequate and effective in improving IBS symptoms also in the long term.

## 1. Introduction

Irritable bowel syndrome (IBS) is a common functional gastrointestinal (GI) disorder, characterized by an alteration of the gut–brain axis. The pathophysiology of IBS is quite complex, involving a combination of motility disturbance, visceral hypersensitivity, altered mucosal and immune function, altered gut microbiota and altered central nervous system processing. Moreover, food plays a not negligible role [1,2].

IBS affects up to 11% of the world’s population, with an increased prevalence in women. Typical IBS symptoms are abdominal pain related to defecation and impaired bowel habits (Table 1). Although it is not a life-threatening disease, it deeply changes the patients’ quality of life. This is also because adequate therapies to treat this syndrome are not yet available and the therapeutic strategy focuses mainly on treating the different symptoms, without a global approach, often obtaining partial and unsatisfactory results [2,3,4,5].

Many patients identify food as a precipitating factor of their symptoms. Consequently, they tend to limit or totally eliminate the intake of certain foods which are considered, wrongly or rightly, the culprit, with the risk of causing nutritional deficiencies [7]. However, it is not clear whether the patients are really able to precisely recognize the foods which trigger their symptoms or if they simply think they are intolerant to some foods, often depicted as dangerous by the media, relatives and friends, e.g., gluten and/or lactose. A relatively new approach to IBS therapy is the restriction of short-chain fermentable oligosaccharides, disaccharides, monosaccharides and polyols (FODMAP), the so-called low-FODMAP diet (LFD) [8].

The “FODMAP” term was coined by the Monash group and presented to the scientific community in a paper concerning a hypothesis (the FODMAP hypothesis) about the pathogenesis of Crohn’s disease [9]. Since then, an LFD has been suggested for many GI disorders, mainly IBS, and also for non-GI disease [8].

FODMAPs are a large class of little or non-digestible carbohydrates, small in size and with high osmotic capacity and high fermentability. They can cause symptoms in IBS patients by increasing small bowel water content and producing colonic gas and short-chain fatty acids (SCFA). SCFA can act on intestinal motility, on water and sodium absorption, and through the modulation of mastocyte activity, even on histamine release and visceral sensitivity [10]. Therefore, reducing the content of the FODMAP diet can be a therapeutic option for IBS patients.

A strict LFD involves a restriction in FODMAP intake for 4–8 weeks, followed by gradual re-introduction according to individual tolerance, thus allowing the personalization of the diet and its use in the long term (Adapted Low-FODMAP Diet, AdLFD). To ensure nutritional adequacy in the long term, it is mandatory that the reintroduction phase is closely monitored by a qualified and skilled nutritionist [11].

As it is an exclusion diet, the critics of the LFD pose doubts regarding its nutritional adequacy and its complexity and efficacy, mainly linked to a supposed poor compliance in the long term [10]. An important issue is the role of FODMAPs as prebiotics: especially through metabolization of FOS (fructooligosaccharides) and GOS (galactooligosaccharides), they favor the growth of beneficial bacteria and increase the production of SCFA, which have an important protective and trophic activity on the colonocytes [8]. A FODMAP reduction, potentially inducing a decrease of fiber intake, could increase the risk of constipation and induce possible changes of the intestinal microbiota with a decrease in the production of SCFA.

An LFD is not only a restriction diet, but it could also be a “diagnostic” tool to test the patient’s tolerance to some foods, enabling the nutritionist and gastroenterologist to eliminate from the diet only the trigger foods causing IBS symptoms. Currently, there are several studies that prove the efficacy of an LFD as a treatment for IBS, although not all are based on randomized, placebo-controlled, double-blinded trials, which are considered the “gold standard” for therapeutic trials. Moreover, most trials are aimed at demonstrating LFD efficacy in the short term and there are very few long-term studies, after the implementation of an AdLFD [12].

The primary endpoint of the present study was the evaluation of the effects of the LFD in the short- and long-term on abdominal symptoms, quality of life, anxious-depressive symptoms, quality of sleep, degree of relief and degree of patient satisfaction in IBS patients.

As secondary endpoints, we also assessed the patients’ adherence to the LFD and AdLFD, their acceptability, the food-related quality of life and the patient’s ability to reliably identify FODMAP foods capable of triggering IBS symptoms.

## 2. Materials and Methods

Subjects meeting Rome IV diagnostic criteria for IBS, who had been referred from the outpatient services of the Gastrointestinal Unit of the University of Pisa, were consecutively enrolled from 1 September 2016 to 31 December 2018 at the Nutritional Outpatient Clinic of the Division of Rheumatology of the University of Pisa.

Patients with known or suspected organic or psychiatric illnesses and those who had taken up to 4 weeks before diagnosis laxatives and/or drugs active on abdominal pain and abdominal swelling/distention were excluded. Routine blood tests, coeliac serology, TSH (Thyroid-stimulating hormone) evaluation, abdominal ultrasound, Lactose breath test, Glucose Breath test, SeHCAT test (75-selenium homocholic acid taurine test) and colonoscopy were performed when indicated to rule out possible organic disease, lactose intolerance, Small Intestinal Bacterial Overgrowth, Bile Acid Diarrhea and/or other organic causes of digestive symptoms.

The study protocol was approved by the Ethical Committee of Pisa (study number 1136/2016) and was carried out in accordance with the Helsinki Declaration (Sixth Revision, Seoul 2008). Signed informed consent was obtained from each participant.

Patients were evaluated before starting the LFD (T0), after 8 weeks of strict LFD (T1) and after the reintroduction phase, when the AdLFD started (T2). After T2, the patients involved in a prospective long-term follow-up were evaluated every six months in order to check symptoms, adherence and compliance to the AdLFD (T3). The data reported here as T3 refer to a medical exam conducted at least 6 months and no more than 24 months after T2.

At each medical exam, each patient underwent the following:

### 2.1. Nutritional Assessment and Anthropometrical Measurements

Detailed personal and behavioral information, medical history and a thorough dietary history were collected by means of a structured interview questionnaire. Anthropometrical measurements consisting of height, weight, upper arm, waist and hip circumferences were performed according to the international criteria [13]. The body mass index (BMI) was calculated for each patient.

### 2.2. BIVA (Bioelectrical Impedance Vector Analysis)

The patients’ body composition and hydration status were evaluated using a Biolectrical Impedance Vector Analysis (BIVA) device (Akern, Florence, Italy; RJL Systems licensee, Clinton Twp, MI, USA) [14,15,16]. BIVA evaluates total body water (TBW), extracellular and intracellular water (ECW, ICW), fat-free mass (FFM), fat mass (FM), body cellular mass (BCM), phase angle (PhA) and basal metabolic rate (BMR). Bioimpedentiometry records the speed and the modification of the current and provides the electrical data, resistance (R) and reactance (Xc), detected by calculating the impedance of the tissues crossed. The dedicated software (BODYGRAM plus) plots the impedance measurements (resistance and reactance) as a vector in a coordinate system [17]. Reference values adjusted for age, BMI and gender are plotted in the so-called tolerance ellipses in the coordinate system [18]. BIVA is an innovative approach, independent of hydration status, which enables a reliable assessment of body composition and hydration in several populations, in all life-cycle stages, and in healthy people as well as in ill subjects [19,20].

In the 8 h prior to the exam, the patients had not drunk alcohol, not performed intense physical activity or had any fever. The patients were placed on their back on a couch in a quiet environment with adequate temperature and humidity for at least 5 min, in order to allow a redistribution of liquids. The measurement was carried out by placing a pair of electrodes on the back of the patient’s right hand and another pair on the back of their right foot (tetrapolar hand–foot technique) [21]. A very low-intensity (400 µÅ) and high-frequency (50 Khz) alternating current was applied to the patient’s body.

### 2.3. Questionnaires

During each medical exam, the patients were invited to fill in the following questionnaires:

1. IBS—Symptom Severity Score (IBS-SSS): this evaluates the severity of abdominal symptoms [22]. It consists of 5 questions that investigate the presence and severity of abdominal pain or discomfort, frequency of abdominal pain, presence and severity of abdominal distension, degree of dissatisfaction with defecatory function and degree of interference of IBS symptoms in work and life habits [3]. Each question is answered by indicating the percentage of the symptomatology on a visual analogue scale (VAS), which generates a score from 0 to 100, where 0 indicates “not at all/absent”, 50 indicates “quite important” and 100 indicates “very important”. The maximum final score, obtained from the sum of the individual values, is 500. A score higher than 300 is a sign of severe symptoms, from 175 to 300 indicates moderate symptoms and between 75 and 175 is a sign of mild symptoms.
2. Bowel habits questionnaire: a ‘‘homemade’’ bowel habits questionnaire evaluating the frequency of (a) straining at defecation, (b) incomplete evacuation, (c) painful defecation, (d) hard stools (Bristol Stool Scale 1–2), (e) watery stools (Bristol Stool Scale 6–7), (f) fragmented defecation, (g) defecatory urgency, (h) incontinence for gas and/or feces, (i) abdominal pain and (j) abdominal bloating, using a scale ranging from 0 (no symptoms) to 4 (symptoms present during >75% of bowel movements or days) [23].
3. SF–36 (Italian version): a questionnaire able to measure the health-related quality of life in the general population [24,25]. It consists of 36 questions divided into 8 sections, each investigating a different aspect of health:
  - Physical functioning (10 questions)
  - Social functioning (2 questions)
  - Role limitations (physical problems) (4 questions)
  - Role limitations (emotional problems) (3 questions)
  - Mental health (5 questions)
  - Vitality (4 questions)
  - Pain (2 questions)
  - General health (5 questions)
  - Health change (1 question)
  From each section, a score on a scale from 0 to 100 is obtained, where 0 is the worst possible health state measured by the questionnaire and 100 is the best possible health state. It is also possible to calculate two summary indexes: one for physical function (PFI) and the other for mental function (MFI), standardized versus a normal value of 50 ± 10.
4. Hospital Anxiety and Depression Scale (HADS): used to investigate the presence and severity of anxiety and depression with a good sensitivity and specificity [26]. It includes 14 questions divided into two groups: 7 questions investigate anxiety symptoms (HADS-A) and 7 assess symptoms of depression (HADS-D). Each question is given a score from 0 to 3, with a possible total score ranging from 0 to 42.
5. Pittsburgh Sleep Quality Index (PSQI): this investigates the quality of sleep and possible sleep disorders over a period of one month. It consists of 19 questions from which 7 overall scores are obtained, each representing a particular aspect of sleep: subjective quality, latency, duration, habitual sleep efficacy, sleep disturbances, use of sleeping medication and daytime dysfunctions. The sum of the scores of these seven components, to which a value from 0 to 3 is attributed, produces the overall result of the PSQI. It can vary from 0 to 21, where a score higher than 5 is considered indicative of sleep disorders [27,28].

At T1 and T3 the following evaluations were also carried out:

1. Degree of relief: this estimates the symptom improvement perceived by the patient compared to T0. The answer is indicated on a 7-point visual analogue scale, where 7 means that the patient feels much worse than at the beginning of the treatment, 4 means nothing has changed and 1 indicates that the patient feels completely relieved, with a remission of symptoms [29,30].
2. Degree of treatment satisfaction: a single question that estimates patient satisfaction with the LFD or AdLFD. The patient is asked to mark the answer on a 10-point visual analogue scale, where 0 indicates that the patient is totally dissatisfied with the diet and 10 indicates completely satisfied [29,30].

At T1 and T3, the following were added:

1. FODMAP Adherence Report Scale (FARS): this evaluates how much the patient adhered to the diet. It consists of 5 questions, each offering five possible answers (always, often, sometimes, rarely and never) to which a score is assigned respectively from 1 to 5, with a maximum score of 25. A total score of at least 20 points (≥80%) is considered as adherence to the diet [31].
2. LFD acceptability questionnaire: composed of 13 items adapted from the nutrition-related QOL (Quality of Life) questionnaire [32]. This investigates the impact of the diet on everyday life. The answers are categorized using 3 possible answers (agree, neutral and disagree) [33] (Table S1 in the Supplementary Materials).
3. Food-related QOL questionnaire: a seven-item questionnaire, based on a 3-point Likert scale (agree, neutral and disagree), investigating the relationship with food and meals [34] (Table S2 in the Supplementary Materials).

At T0 and T2, the following was also evaluated:

1. Perception of “trigger” foods: at T0, the patients were asked if there were some FODMAP groups that they could identify as trigger food, e.g., causing bloating, pain and/or impairing the quality of their defecation (bowel movements and stool consistency). Data were compared with the results obtained at the end of the reintroduction period (T2) to evaluate the concordance in detecting the FODMAP foods able to provoke their symptoms.

### 2.4. Enrollment (T0)

At T0, a strict LFD, where high-FODMAP foods were replaced by low-FODMAP foods, was prescribed by the nutritionist, according to Shepard and Gibson, and recommended for 8 weeks [35]. It was adapted to the Italian dietary habits to ensure both acceptability by the patients and nutritional adequacy (Table 2). To calculate the content of FODMAPs in the diet, we applied the cutoff values to individual food (per serving) by means of the published FODMAP table content [36,37,38,39].

The composition of the diet was optimized for individual patients in order to both strengthen adherence to the diet and guarantee nutritional adequacy in terms of proteins, carbohydrates, lipids, minerals and vitamins. General dietary advice was also given, such as slow chewing, drinking at least 1.5 L of water a day and carefully reading food labels on foods. 

Every 2 weeks, the nutritionist contacted the patients by telephone in order to resolve any problems related to dietary management. The nutritionist could also always be contacted by patients via e-mail.

### 2.5. First Check-Up (T1)

After 8 weeks, the patients were recalled for a gastroenterological and nutritional check-up (T1). Anthropometric measurements and BIVA were performed again. Patients were asked to complete the same questionnaires they had completed at T0, and also the questionnaires on adherence, acceptability and food-related QOL. They were also asked to answer the questions about the degree of relief and the satisfaction with the diet.

During the medical check-up, the re-introduction of foods containing FODMAPs was explained (Table 3 reports foods used as “trigger foods” and the suggested amount). The FODMAPs were reintroduced, one at a time), for four days, according to Shepard and Gibson, to determine which contributed to the symptoms, with a break between one food and another (1 or 2 weeks), according to a precise design depending on the symptoms produced by the food [35]. If there were no symptoms or only mild ones, the break lasted 1 week, otherwise it was extended to 2 weeks. The aim was to identify the foods able to provoke digestive symptoms. The patient was invited to fill in a 4-day diary in which they had to mark the presence and the severity of bloating and abdominal pain (using a 5-point scale), the number of defecations and the fecal consistency.

### 2.6. Second Check-Up (T2)

After the end of the reintroduction phase, the patients were called back again for a gastroenterological and nutritional check-up. Anthropometric measurements and BIVA were carried out again and the perception of trigger foods was evaluated on the basis of the above criteria. The score > 2 of bloating or pain or an increase of at least 1 point in comparison with the previous period, and/or changes in defecation frequency, and/or Bristol Scale outside the range of normality, and/or the patient’s answer “yes” to the question “Did the reintroduction of this food deeply increase your digestive symptoms and/or impair the quality of your defecation?” were considered as the parameters useful to identify the food as a “trigger food” [35]. The target was to identify the foods containing FODMAPs that the patient was able to consume without having an exacerbation of symptoms, enabling the nutritionist to suggest a long-term and less strict diet (AdLFD), customized for each patient on the basis of different FODMAP intolerance.

### 2.7. Third Check-Up (T3)

After a minimum period of 6 months from the beginning of the AdLFD, and every six months, patients were invited to a new gastroenterological and nutritional evaluation (T3). During the visit, anthropometric measurements and BIVA analysis were conducted again and the patients were invited to fill in the questionnaires.

### 2.8. Data Analysis and Statistical Tests

Quantitative data were described with mean and standard deviation (SD). Normality of the quantitative variables’ distributions was assessed with the Kolmogorov–Smirnov test. To analyze quantitative variables, the t-test for independent samples was used. To compare repeated measures (T0, T1, T2 and T3), analysis of variance (ANOVA) for repeated measures was applied, followed by multiple comparisons with the Bonferroni method. Agreement analysis was performed by the Cohen test and results were expressed by Cohen’s K. Significance was fixed at 0.05. All analyses were carried out by Excel and SPSS v.26 technology (IBM, New York, NY, USA).

## 3. Results

Seventy-three patients were recruited (64 female; F), 9 male; M); Mean age 45.27 ± 11.94 years).

At T0, 30/73 patients (41%) had IBS with diarrhea (IBS-D), 20/73 (27.4%) IBS with constipation (IBS-C) and 23/73 (31.6%) IBS with mixed bowel (IBS-M), while at T3, 17/41 (41.5%) had IBS-D, 13/41 (31.7%) had IBS-C and 11/41 (26.8%) had IBS-M. No significant difference between the subgroup composition at T0 and T3 was observed.

Sixty-eight patients (61 F, 7 M; Mean age 45.28 ± 12.08) completed the 8-week LFD and were evaluated at T1 starting the reintroduction phase. Fifty-nine patients (55 F, 4 M; Mean age 45.02 ± 12.25 years) were evaluated at T2 and started the AdLFD. Forty-one patients (37 F, 4 M; Mean age 45.63 ± 11.68 years) were evaluated at T3.

**Figure 1:**
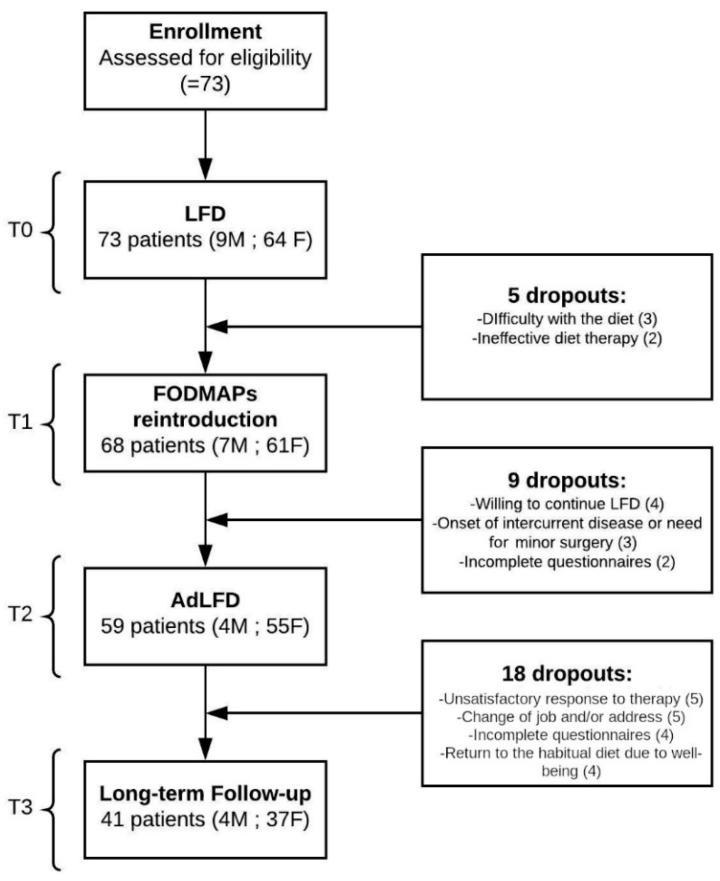
Study design. LFD: Low-FODMAP Diet, FODMAP: fermentable oligosaccharides, disaccharides, monosaccharides and polyols, AdLFD: Adapted Low-FODMAP Diet. The boxes on the right show the number of dropouts and the reasons for them are reported at each time.

The mean follow-up was 12.7 ± 10.0 months (6–24 months). The design of the study and the reasons for drop-out at each time are reported in Figure 1. Seventy-three patients were recruited, of which 41 were fully evaluated longitudinally, so the reported results refer to the 41 patients evaluated at T3.

Because no significantly different rate of response was observed at T1, T2 and T3 between the different IBS subgroups (IBS-D, IBS-C, IBS-M), apart from a lower frequency of loose stools and defecatory urgency in IBS-D, the results reported in the paper are those of the whole group of IBS patients.

### 3.1. BIVA and Anthropometrical Measurements

No significant differences were observed for anthropometric data and BIVA parameters before starting the LFD (T0), at the end of the 8-week LFD (T1), after the reintroduction phase (T2) and at the follow-up (T3), showing that the LFD and AdLFD did not change body composition, hydration status and phase angle (Table 4; see also Table S3 in the Supplementary Materials for complete Anthropometric data).

### 3.2. IBS-SSS

**Figure 2:**
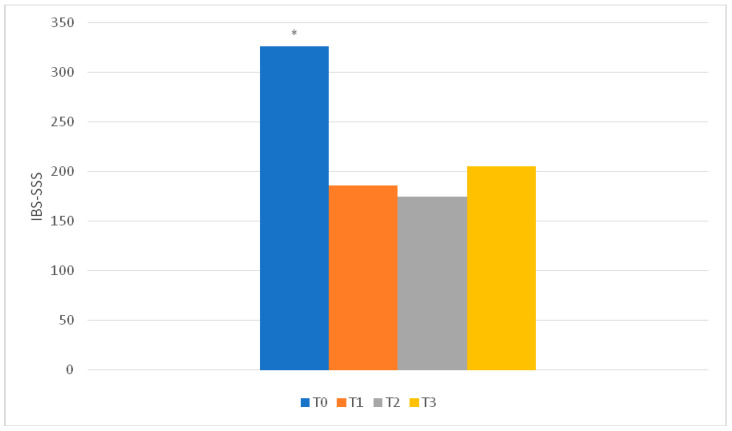
IBS-SSS (IBS—Symptom Severity Score) total score results. * T0 vs others: *p* < 0.001. No significant difference was observed between T1, T2 and T3. The reported results refer to the 41 patients evaluated at T3.

Severity of abdominal pain, days with abdominal pain, severity of abdominal distension, bowel habit dissatisfaction and IBS interference with lifestyle habits improved, as well as total score, at T1 and T2 compared to T0. These results were maintained at T3. At T3, no difference in comparison with T1 and T2 was observed, confirming the long-term efficacy of the diet (Figure 2; Table S4 in the Supplementary Materials).

At T1, 34/41 patients had a reduction of >50 points and a further 5 patients reached a reduction of >50 points at T2: as a whole, 39/41. One patient did not report any improvement and one other patient, with starting IBS-SSS < 175, reported an improvement of <50 points. At T3, 34/41 patients continued to report a reduction of >50 points.

### 3.3. Bowel Habits

**Figure 3:**
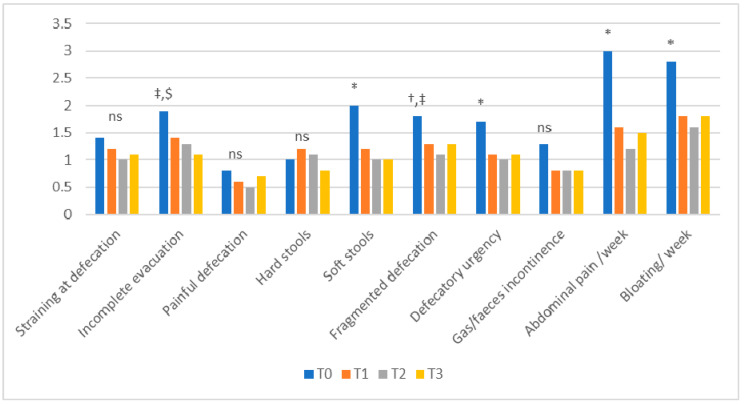
Bowel habits results. * T0 vs other: *p* < 0.05; † T0 vs T1: *p* < 0.05; ‡ T0 vs T2: *p* < 0.05; $ T0 vs T3: *p* < 0.05; ns: not significant. The reported results refer to the 41 patients evaluated at T3.

The bowel habit questionnaire showed a significant improvement at T1, T2 and T3 compared to T0 for watery stools, defecatory urgency, abdominal pain and bloating. At T2 and T3, compared to T0, there was improvement regarding fragmented defecation and gas/feces incontinence, and at T3 compared to T0, for the feeling of incomplete evacuation. These data confirm that the bowel habit improvement was also maintained in the follow-up (T3) (Figure 3).

### 3.4. SF–36

**Figure 4:**
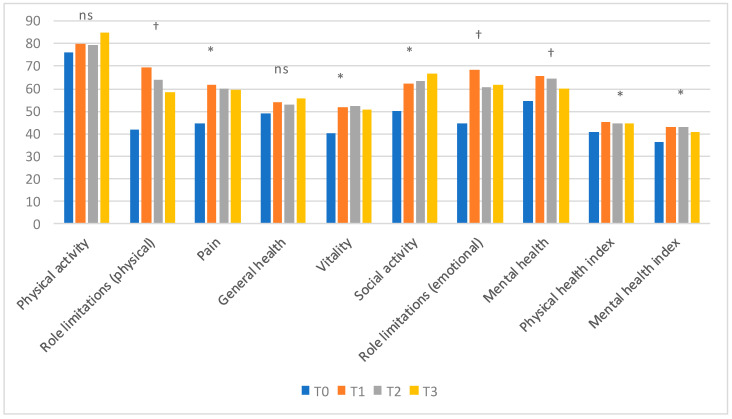
SF-36 results. * T0 vs other: *p* < 0.05; † T0 vs T1: *p* < 0.05; ns: not significant. The reported results refer to the 41 patients evaluated at T3.

The evaluation of the quality of life showed that there was a significant improvement in many of the parameters examined. Pain, vitality and social functioning improved significantly at T1, T2 and T3 compared to T0. Role limitations (physical and emotional) and mental health significantly improved only at T1 with respect to T0. Physical functioning and general health did not differ at any time. Physical and mental health indexes, which are the summary indexes, significantly improved at T1, T2 and T3 compared to T0 (Figure 4).

### 3.5. Hospital Anxiety and Depression Scale (HADS)

The HADS questionnaire showed that anxiety symptoms significantly improved at T1, T2 and T3 compared to T0 (*p* < 0.001). There was also a significant improvement in depressive symptoms at T1, T2 and T3 in comparison with T0 (*p* < 0.05) (Table 5).

### 3.6. Pittsburgh Sleep Quality Index (PSQI)

At T0, the PSQI score was 8.29 ± 4.40, showing an insufficient quality of sleep in our group of IBS patients. The scores at T1 (7.30 ± 3.56), T2 (6.68 ± 3.51) and T3 (7.92 ± 5.82) slightly, but not significantly, improved in comparison with T0.

### 3.7. Evaluation of the Degree of Relief and Degree of Treatment Satisfaction

The degree of relief, evaluated on a 7–0 scale, showed a clear improvement in symptoms at T1 (1.5 ± 0.9), confirmed at T3 (1.2 ± 1.3) (ns). The degree of treatment satisfaction, assessed on a 0–10 scale, was 8.5 ± 1.3 at T1 and 8.0 ± 1.9 at T3 (ns).

### 3.8. FODMAP Adherence Report Scale (FARS)

The patients showed a good adherence to the diet at T1 (23.1 ± 2.4). Adherence assessed at T3 appeared to have decreased just below the cut-off value (19.1 ± 2.8; *p* < 0.0001).

### 3.9. Acceptability of the Diet

During the LFD, 65.8% of the patients and 51.2% during the AdLFD reported that they spent more time for shopping compared with their habitual diet (HD) (*p* < 0.00005 and *p* < 0.003, respectively). 60.9% during the LFD and 39% during the AdLFD reported that they spent more time for cooking (*p* < 0.0001 and *p* < 0.01, respectively). During the AdLFD, there was a slight, but not significant, reduction of time spent for shopping and cooking compared with the LFD. Patients reported that both the LFD (75.6%) and AdLFD (58.5%) were more expensive in comparison with their HD (*p* < 0.00005, and *p* < 0.0003, respectively), whereas no differences between the LFD and AdLFD were found even if the AdLFD was judged to be slightly, but not significantly, cheaper than the LFD. During the LFD, 85.2% of the patients and 58.5% during the AdLFD reported increased difficulty eating out at restaurants compared with the HD (*p* < 0.00001 and *p* < 0.03, respectively). During both the LFD and AdLFD, 76.6% of the patients found increased difficulty in eating out at friends (*p* < 0.00001) and 65.8% found more difficulty eating during travel (*p* < 0.0002). Patients found the LFD (24.3%) and AdLFD (29.2%) less tasty and enjoyable than their HD (*p* < 0.04 and *p* < 0.02, respectively).

### 3.10. Food-Related QOL

Concerning the food-related QOL, patients suggested that the conditions of their life regarding food were better both with the LFD (51.2%) and the AdLFD (56.1%) in comparison with their HD (15.0%) (*p* < 0.01 and *p* < 0.007, respectively). Patients were more satisfied with food and meals in daily life during the LFD (70.7%) and AdLFD (70.7%) compared to the HD (45.0%) (*p* < 0.05 for both comparisons). The wish that their meals were a much more pleasant part of their life was significantly higher with the HD (60.0%) compared to the LFD (29.2%) and AdLFD (33.9%) (*p* < 0.03 and *p* < 0.05, respectively).The feeling of “seeing problems with food” significantly decreased during the LFD (9.8%) and AdLFD (14.6%) in comparison with the HD (60.3%) (*p* < 0.0003 and *p* < 0.003, respectively).

### 3.11. Perception of Trigger Foods

**Figure 5:**
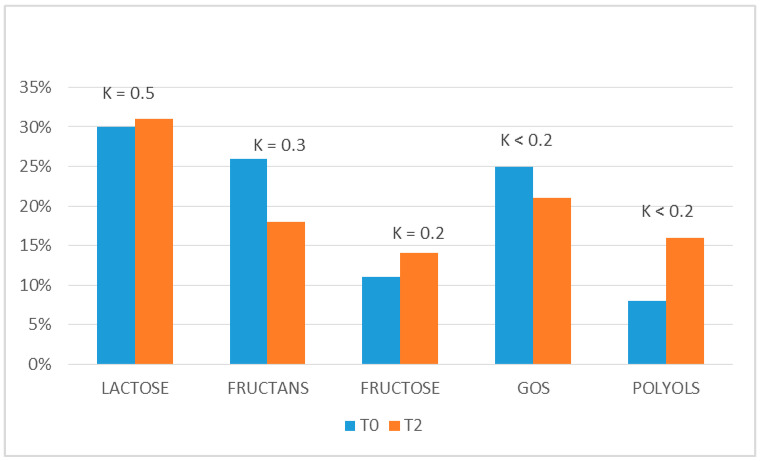
Perception of trigger foods. The concordance (expressed by Cohen’s K) between the perception of which groups of FODMAP were able to trigger IBS symptoms before starting LFD (T0) and after the end of the reintroduction phase (T2). The reported results refer to the 41 patients evaluated at T3.

All the patients identified at least one FODMAP food as a symptom trigger during the clinical history at T0. This perception was compared with the results obtained at the end of the reintroduction phase (T2), used to plan the AdLFD, and they were quite different. Indeed, the concordance between T0 and T2 (expressed by Cohen’s k) was moderate (K = 0.5) for lactose, fair for fructans (K = 0.3) and poor for polyols (K < 0.2), fructose (K < 0.2) and galactans (K < 0.2). This expresses a high variability of perception of “trigger foods” between T0 and T2 (Figure 5).

## 4. Discussion

In recent years, the LFD has gained increasing popularity among gastroenterologists and patients. Even if it cannot be considered a panacea for all patients affected with GI disturbances, there are now several studies proving its efficacy [8]. Indeed, a recent systematic review provided evidence that an LFD is effective in improving IBS symptoms [12]. Even if the evidence is of very low-quality according to the GRADE system, the LFD was the most effective among the dietary interventions suggested for treating this syndrome [40]. The different types of comparator groups used in the various trials, the difficulty of finding a real sham diet for the placebo group and the different parameters used for evaluating the improvement are only some of the potential sources of bias which could explain the relatively low level of evidence. These problems could be solved by well-designed randomized controlled trials (RCTs) using greater numbers of patients and similar comparator groups. However, at least in the current economic situation, it is not easy to find subjects willing to finance such research projects. Moreover, some doubts and concerns raised about the LFD remain to be clarified [8]. The critics of the LFD state that it is effective only in the short term, is nutritionally inadequate, and is complex and difficult to teach and learn, difficult to continue and potentially expensive. These issues and limitations are undoubtedly amplified in patients who follow the LFD without professional advice, because the role of a skilled nutritionist is essential [11]. We think that the present paper could contribute to shedding light on many of these issues.

The LFD was effective in reducing IBS symptoms in our group of patients, not only after the LFD period (T1) but also at T3 (the follow-up visit, not less than six months after starting the AdLFD), as shown by the IBS-SSS. Indeed, when evaluating the number of patients showing a reduction of more than 50 points of IBS-SSS at T3, it was similar to that observed at T1 and a little lower, but not significantly, than that observed at T2. These data confirm the good results obtained by some previous studies [32,41]. It is also worth underlining that the LFD improved bowel habits, mainly defecatory urgency, fragmented defecation and the feeling of incomplete evacuation. Moreover, also fecal consistency (reducing the presence of watery stools) improved without increasing the presence of hard stools, which is often considered an inevitable consequence of the reduction in fiber intake if wholegrain wheat products or high-FODMAP fruit and vegetables are not replaced with suitable low-FODMAP alternatives. We think that the prompt and careful suggestions of a skilled nutritionist prevented the onset of this possible drawback in our IBS patients.

Even considering all the dropouts as non-responders, although this is not the case given that four patients included in the dropout group did not want to stop the LFD because it was highly effective on their symptoms, the percentage of patients who judged the LFD and AdLFD to be effective in the follow-up is about 50%. This percentage is coherent with data available in previously published papers regarding short-term studies (50–76%) and does not differ from the results obtained in many trials also with new and expensive drugs for IBS [42,43].

The effect on GI symptoms is likely the most important factor affecting the improvement of the quality of life, as shown by the SF-36. The patients reported a significant improvement in many of the evaluated parameters in the medium and long term. In particular, the “summary” indexes (Physical and Mental Index) had improved at T1 and remained improved, without any significant difference, also at T2 and T3. Also, anxiety and depression, as shown by the HADS questionnaire, had significantly improved at T1 and also at T3. The PSQI, on the contrary, shows only a trend, not statistically significant, towards an improvement. This could be due to the fact that, as IBS symptoms are mainly present during the day and disappear during the night, the digestive symptom improvement may not have affected sleep quality. However, the degree of relief and the degree of satisfaction with the diet, which were constantly high at T1, T2 and T3, are the “litmus test” of the data reported above, confirming the long-term efficacy of this dietary approach in our group of patients.

The nutritional adequacy of an LFD is another frequently raised concern. In the present study, this issue was investigated by means of anthropometric measures, mainly BIVA. BIVA enables evaluation of body composition and hydration. It also provides an interesting parameter, the Phase Angle (PhA), which changes quickly after only 1 week of dietary modifications, making BIVA a very useful tool to check the nutritional status of patients [44]. We did not find any significant difference for any of the evaluated anthropometric data and BIVA parameters by comparing the results at any time of the study. Particularly, BIVA revealed no change in fat-free mass, fat mass, intracellular or extracellular water, thus showing the nutritional adequacy of the 8-week LFD and also of the AdLFD. These results are corroborated by the unchanged PhA values and are coherent with the results previously published in a paper by our group [45]. Up to now, these are the only published data on the nutritional monitoring of the LFD in IBS patients using this kind of analysis.

LFD adherence and acceptability are two other important matters of debate. If a diet is too “strict” and/or difficult to follow and/or too expensive, even if it is very effective, the patients tend to abandon it in the medium–long term. The present results testify that IBS patients had excellent adherence at T1 but in the follow-up (T3), they tend to be less adherent, even if the improvement in IBS symptoms and bowel habits still remains. The length of the follow-up could have influenced this result. Moreover, the higher adherence observed at T1 may be due to the fact that patients are more loyal during the first phase of the LFD, the “strict phase”, whereas, in the long term, probably because they feel better, they tend to reintroduce many forbidden foods, lowering their adherence. Effectively, any chronic therapy, even if it is obtaining good results, tends to lose adherence over time [46]. In addition, it has to be underlined that a further contribution to a numerical result of lower adherence could be due to the patients who preferred to continue the “strict” LFD for fear of hindering the improvement made. Indeed, four of our patients were considered dropouts because they wanted to follow the “strict” LFD instead of starting the AdLFD.

Regarding LFD acceptability, the patients had some difficulties about the time spent for food shopping and cooking. Moreover, they found difficulties in eating at restaurants or during traveling compared to their habitual diet. We may speculate that an LFD poses some difficulties, especially at the beginning of the treatment during the first phase of strict restriction. These difficulties tend to be less evident and/or to be overcome during the AdLFD. Our data confirm that both the LFD and AdLFD are not “easy” diets, but they require the constant interplay with a skilled nutritionist, at least in their first phases. However, when they are carried out in the correct way, they do not adversely affect the food-related QOL. On the contrary, they improve the patients’ relationship and satisfaction with food and decrease the feeling of food as a source of problems.

The patients’ reliability in detecting the real FODMAP foods able to provoke their symptoms was not high. Indeed, the molecule most frequently identified as responsible for the onset of the symptoms was lactose, but the concordance between T0 and T2 was only moderate. For the other FODMAPs, the ability to perceive them as triggers during the clinical history of the starting visit (T0) was even lower. Indeed, at T0, many patients indicate fructans as “dangerous” foods, but this impression was not confirmed by the data of the reintroduction phase (T2). This is plausible because foods such as bread and pasta, which are the main source of fructans and are an important component of the Mediterranean diet, are often the victims of many advertising media campaigns and many people self-diagnose a non-coeliac gluten sensitivity and start a gluten-free diet. On the basis of the present results, which should be confirmed by larger multi-center studies involving, if possible, a re-challenge phase, we would suggest that patients should not rely exclusively on their own perceptions. They should be guided by a skilled nutritionist so as to reliably identify foods able to provoke their abdominal symptoms.

The present study has some limitations. First of all, the number of patients enrolled in the follow-up is not large. However, the length of the follow-up itself is not short if compared with other LFD studies and we are confident that the amount of data collected investigating different clinical features deserves some interest [31,32,41,46,47,48]. Moreover, gut microbiota was not characterized. Some authors maintain that it could play an important role both in the pathogenesis of the disease and in the effects of an LFD, even if studies investigating gut microbiota and its metabolic products in IBS patients after LFD have shown mixed, and sometimes conflicting, results [49]. Moreover, Harvie et al., examining gut microbiota after the reintroduction period, found that the LFD did not reduce microbiota diversity [46]. In addition, the present study, similarly to most studies investigating the LFD in IBS, is not a randomized placebo-controlled study. This clearly lowers the level of evidence of our results but, as stressed by many authors, the difficulty of finding a real sham diet for the placebo-group is a very common and difficult condition to overcome, particularly when evaluating such a well-known nutritional approach like the LFD [8]. Finally, the number of dropouts is not negligible, but this group also included four patients who preferred continuing their strict LFD because it had been very effective and they were worried that an AdLFD could limit the progress of their symptoms, and four patients who preferred to start their previous HD again because they felt well.

## 5. Conclusions

The present results confirm the efficacy of an LFD in improving the GI symptoms of IBS patients, their anxiety and depressive symptoms and their quality of life, not only in the short term, during the “strict” phase, but also in the long term, and during the follow-up, when a more relaxed diet (i.e., AdLFD) is prescribed. Moreover, even if some problems regarding acceptability and adherence are reported, the present data support the nutritional safety of both the LFD and AdLFD and their role in inducing a better approach by the patients to food, no longer seen as a cause of illness. We are confident that the length of follow-up could be reasonably sufficient to consider that these effects are not due to a mere “placebo effect”. Finally, the pivotal role of a skilled nutritionist, able to explain the nature and the aim of the diet, ensuring nutritional adequacy and favoring patients’ compliance, has to be stressed.

## Figures and Tables

**Table 1:** Rome IV diagnostic criteria for irritable bowel syndrome (IBS).

Recurrent abdominal pain, at least 1 day per week in the last 3 months, associated with 2 or more of the following characteristics: Related to defecation Associated with a change in stool frequency Associated with a change in stool appearance
Criteria fulfilled with symptom onset in the last 3 months with an onset at least 6 months prior to diagnosis [6].

**Table 2:** Mean dietary intake of patients at baseline (habitual diet) and on a low-FODMAP (fermentable oligosaccharides, disaccharides, monosaccharides and polyols diet) (LFD). No significant difference in energy and macronutrients content were found between the habitual diet and LFD and AdLFD (Adapted LFD). The reported results are referring to the 41 patients evaluated at T3. ns = not significant.

	Habitual Diet	LFD	AdLFD	*p*-Value
**Energy (kcal)**	1996 ± 541	1957 ± 459	1972 ± 510	ns
**Proteins (g)**	90.3 ± 43.3	88.4 ± 47.2	91.4 ± 44.7	ns
**Fats (g)**	72.2 ± 24.1	69.5 ± 18.2	71.5 ± 20.3	ns
**Carbohydrates (g)**	249 ± 41	254 ± 59	252 ± 60	ns
**Dietary fibers (g)**	19.8 ± 8.3	18.4 ± 7.2	20.3 ± 9.1	ns
**Calcium (mg/d** **ay** **)**	910 ± 550	870 ± 520	970 ± 580	ns
**Iron (mg/d** **ay** **)**	8.7 ± 4.2	8.6 ± 4.1	9.1 ± 4.5	ns
**Zinc (mg/day)**	11.3 ± 4.4	11.1 ± 4.5	11.3 ± 4.5	ns
**Magnesium (mg/day)**	420 ± 90	390 ± 110	430 ± 107	ns
**Sodium (g/day)**	2.5 ± 1.8	2.3 ± 1.7	2.3 ± 1.8	ns
**Potassium (g/day)**	4.1 ± 1.9	3.9 ± 1.8	3.9 ± 1.8	ns
**Phosphorus (mg/day)**	1863 ± 630	1932 ± 710	1879 ± 693	ns

**Table 3:** FODMAP reintroduction: the foods suggested as trigger foods for each FODMAP group and their suggested amount are reported.

**Fructans**	50 g of wheat bread or pasta or 1 clove of garlic or ¼ onion
**Lactose**	125 mL of milk
**Fructose**	2 teaspoons of honey
**Polyols**	mushrooms (100 g fresh or 10 g dried) or 2 dried apricots
**Galactans**	lentils or legumes (100 g cooked or 30 g dried)

**Table 4:** Biolectrical Impedance Vector Analysis (BIVA) parameters and BMI. TBW: Total Body Water, ECW: Extracellular Water, ICW: Intracellular Water, FFM: Fat-Free Mass, FM: Fat Mass, BCM: Body Cell Mass, PhA: Phase Angle, BMR: Basal metabolic rate, BMI: Body Mass Index. ns = not significant. The reported results refer to the 41 patients evaluated at T3.

BIVA	T0	T1	T2	T3	*p*-Value
TBW (L/m)	20.9 ± 2.6	20.8 ± 2.7	20.9 ± 2.6	20.7 ± 2.6	ns
ECW (%)	0.5 ± 0.0	0.5 ± 0.0	0.5 ± 0.0	0.5 ± 0.0	ns
ICW (%)	0.5 ± 0.0	0.5 ± 0.0	0.5 ± 0.0	0.5 ± 0.0	ns
FFM (kg/m)	28.5 ± 3.6	28.4 ± 3.6	28.6 ± 3.6	28.5 ± 3.2	ns
FM (kg/m)	13.0 ± 5.8	12.8 ± 5.9	12.8 ± 6.6	12.9 ± 6.6	ns
BCM (kg/m)	14.4 ± 2.6	14.5 ± 3.2	13.9 ± 2.0	13.9 ± 2.7	ns
PhA (°)	5.2 ± 0.6	5.2 ± 0.6	5.1 ± 0.5	5.3 ± 0.4	ns
BMR (kcal)	1425.6 ± 119.0	1420.1 ± 118.9	1416.7 ± 117.5	1417.7 ± 117.9	ns
BMI (kg/m^2^)	25.0 ± 4.2	24.8 ± 4.8	24.8 ± 4.9	24.8 ± 4.8	ns

**Table 5:** Hospital Anxiety and Depression Scale (HADS) at T0, T1, T2 and T3. The reported results refer to the 41 patients evaluated at T3. A: Anxiety, D: Depression.

HADS	T0	T1	T2	T3	*p*-Value
**HADS-A**	9.5 ± 4.4	6.4 ± 4.0	6.8 ± 4.4	7.1 ± 4.4	T0 vs others: *p* < 0.001
**HADS-D**	6.8 ± 4.3	5.2 ± 3.9	5.3 ± 4.2	5.2 ± 3.8	T0 vs others: *p* < 0.05

## Supplementary Materials

The following are available online at https://www.mdpi.com/2072-6643/12/8/2360/s1: Table S1: Dietary Acceptability Questionnaire, Table S2: Food-related QOL questionnaire, Table S3: Anthropometric data of IBS patients at T0, T1 and T3, Table S4, IBS-Symptom Severity Score results at T0, T1, T2 and T3.
