# Supplementary material for: A Low-FODMAP Diet for Irritable Bowel Syndrome: Some Answers to the Doubts from a Long-Term Follow-Up
Source: Nutrients. 2020 Aug 7;12(8):2360. doi: 10.3390/nu12082360 (PMC7468924; doi:10.3390/nu12082360)
Supplement: Supplementary file 1 [file nutrients-12-02360-s001.pdf]

SUPPLEMENTARY FILES

Table S1: Dietary Acceptability Questionnaire:

1. I find it easy to buy suitable foods for my current diet at my normal supermarkets or shops

- □ Agree
- □ Neutral
- □ Disagree

2. I am able to buy foods suitable for my current diet at my normal supermarkets or shops

- □ Agree
- □ Neutral
- □ Disagree

3. I use high street/online speciality shops (e.g., health food shops) to buy food for my current diet

- □ Agree
- □ Neutral
- □ Disagree

4. It takes extra time to shop for my current diet

- □ Agree
- □ Neutral
- □ Disagree

5. It takes extra time to cook for my current diet

- □ Agree
- □ Neutral
- □ Disagree

6. I find food labeling is adequate to allow me to confidently choose suitable foods

- □ Agree
- □ Neutral
- □ Disagree

7. The cost of my current diet is more expensive

- ☐ Agree
- ☐ Neutral
- ☐ Disagree

8. Does eating out at restaurants make it more difficult for you to follow your current diet?

- ☐ Agree
- ☐ Neutral
- ☐ Disagree

9. Does eating out with friends/family make it more difficult for you to follow your current diet?

- ☐ Agree
- ☐ Neutral
- ☐ Disagree

10. Does travel (overseas/UK) make it more difficult for you to follow your current diet?

- ☐ Agree
- ☐ Neutral
- ☐ Disagree

11. Overall, I find my current diet tasty and enjoyable

- ☐ Agree
- ☐ Neutral
- ☐ Disagree

12. I can incorporate my current diet easily into my life

- ☐ Agree
- ☐ Neutral
- ☐ Disagree

13. My current dietary needs have created stress with my family/friends

- ☐ Agree
- ☐ Neutral
- ☐ Disagree

Table S2: Food-related quality of life questionnaire:

1. Food and meals are positive elements of my life

- ◻ Agree
- ◻ Neutral
- ◻ Disagree

2. I am generally pleased with my food

- ◻ Agree
- ◻ Neutral
- ◻ Disagree

3. My life in relation to food and meals is close to my ideal

- ◻ Agree
- ◻ Neutral
- ◻ Disagree

4. With regard to food, the conditions of my life are excellent

- ◻ Agree
- ◻ Neutral
- ◻ Disagree

5. Food and meals give me satisfaction in daily life

- ◻ Agree
- ◻ Neutral
- ◻ Disagree

6. I wish my meals were a much more pleasant part of my life

- ◻ Agree
- ◻ Neutral
- ◻ Disagree

7. When I think of my next meal, I only see problems, obstacles and disappointments

- ◻ Agree

□	Neutral
□	Disagree

Table S3: Anthropometric data of IBS patients at T0, T1 and T3. No significant difference was observed. The reported results are referred to the 41 patients evaluated at T3.

	T0	T1	T3	p-value
**Height (cm)**	167.0 ± 1.0	167.0 ± 1.0	167.0 ± 1.0	ns
**Weight (kg)**	70.6 ± 15.6	69.4 ± 15.5	69.8 ± 15.5	ns
**BMI (kg/m2)**	25.0 ± 4.2	24.8 ± 4.8	24.8 ± 4.8	ns
**Wrist circumference (cm)**	16.1 ± 1.3	15.4 ± 3.3	15.8 ± 2.5	ns
**Waist circumference (cm)**	80.0 ± 12.1	79.6 ± 12.3	79.3 ± 12.2	ns
**Hip circumference (cm)**	101.3 ± 9.8	100.6 ± 9.9	101.1 ± 9.9	ns
**Arm circumference (cm)**	27.6 ± 3.1	27.8 ± 3.1	27.8 ± 3.2	ns
**BMR (kcal/die)**	1425.6 ± 119.0	1420.1 ± 118.9	1417.7 ± 117.9	ns

Table S4: IBS-Symptom Severity Score results at T0, T1, T2 and T3. The reported results are referred to the 41 patients evaluated at T3.

IBS-SSS	T0	T1	T2	T3	p-value
**Abdominal pain severity**	58.3 ± 23.4	29.3 ± 24.0	26.0 ± 22.5	33.2 ± 22.7	T0 vs others: $p<0.001$
**Abdominal pain frequency**	64.5 ± 29.6	27.1 ± 23.6	24.2 ± 22.6	28.5 ± 30.8	T0 vs others: $p<0.001$
**Abdominal distension severity**	61.7 ± 23.6	33.4 ± 24.7	31.7 ± 27.5	40.8 ± 24.5	T0 vs others: $p<0.001$
**Dissatisfaction with defecatory function**	71.2 ± 30.3	51.8 ± 34.9	51.7 ± 34.2	55.2 ± 30.3	T0 vs T1: $p<0.05$

					T0 vs T2: p<0.05
**Interference of IBS symptoms with life habits**	70.5 ± 248	44.5 ± 31.5	46.1 ± 31.0	49.8 ± 32.4	T0 vs others: p<0.001
**IBS-SSS total**	326.2 ± 88.7	185.9 ± 97.0	174.8 ± 98.1	205.3 ±106.2	T0 vs others: <0.001
